# Identification of Muscle-Specific MicroRNAs in Serum of Muscular Dystrophy Animal Models: Promising Novel Blood-Based Markers for Muscular Dystrophy

**DOI:** 10.1371/journal.pone.0018388

**Published:** 2011-03-30

**Authors:** Hideya Mizuno, Akinori Nakamura, Yoshitsugu Aoki, Naoki Ito, Soichiro Kishi, Kazuhiro Yamamoto, Masayuki Sekiguchi, Shin'ichi Takeda, Kazuo Hashido

**Affiliations:** 1 Administrative Section of Radiation Protection, National Institute of Neuroscience, National Center of Neurology and Psychiatry, Kodaira, Tokyo, Japan; 2 Department of Molecular Therapy, National Institute of Neuroscience, National Center of Neurology and Psychiatry, Kodaira, Tokyo, Japan; 3 Department of Degenerative Neurological Diseases, National Institute of Neuroscience, National Center of Neurology and Psychiatry, Kodaira, Tokyo, Japan; 4 Department of System Neuroscience, Medical Research Institute, Tokyo Medical and Dental School University Graduate School, Tokyo, Japan; 5 Department of Biological Information, Tokyo Institute of Technology, Yokohama, Japan; French National Center for Scientific Research - Institut de biologie moléculaire et cellulaire, France

## Abstract

Duchenne muscular dystrophy (DMD) is a lethal X-linked disorder caused by mutations in the *dystrophin* gene, which encodes a cytoskeletal protein, dystrophin. Creatine kinase (CK) is generally used as a blood-based biomarker for muscular disease including DMD, but it is not always reliable since it is easily affected by stress to the body, such as exercise. Therefore, more reliable biomarkers of muscular dystrophy have long been desired. MicroRNAs (miRNAs) are small, ∼22 nucleotide, noncoding RNAs which play important roles in the regulation of gene expression at the post-transcriptional level. Recently, it has been reported that miRNAs exist in blood. In this study, we hypothesized that the expression levels of specific serum circulating miRNAs may be useful to monitor the pathological progression of muscular diseases, and therefore explored the possibility of these miRNAs as new biomarkers for muscular diseases. To confirm this hypothesis, we quantified the expression levels of miRNAs in serum of the dystrophin-deficient muscular dystrophy mouse model, *mdx*, and the canine X-linked muscular dystrophy in Japan dog model (CXMD_J_), by real-time PCR. We found that the serum levels of several muscle-specific miRNAs (miR-1, miR-133a and miR-206) are increased in both *mdx* and CXMD_J_. Interestingly, unlike CK levels, expression levels of these miRNAs in *mdx* serum are little influenced by exercise using treadmill. These results suggest that serum miRNAs are useful and reliable biomarkers for muscular dystrophy.

## Introduction

Duchenne muscular dystrophy (DMD) is a lethal X-linked disorder caused by mutations in the *dystrophin* gene, which encodes a cytoskeletal protein, dystrophin[1]. The absence of dystrophin results in progressive degeneration of skeletal and cardiac muscle with fibrotic tissue replacement, fatty infiltration, and subsequent early death by respiratory or heart failure[2], [3]. Creatine kinase (CK) is an enzyme related to energy metabolism present in various types of cells[4]. CK is commonly used as a blood-based biomarker for muscular dystrophy to evaluate the level of muscle damage and necrosis, and the efficacy of potential therapies, but it is not always reliable since it is easily affected by stress to the body, such as exercise[5], [6], [7]. Other markers for muscular dystrophy, such as myoglobin, aldolase or lactate dehydrogenase, also have the same problem. Therefore, more reliable biomarkers of muscular dystrophy have long been desired.

MicroRNAs (miRNAs) are small, ∼22 nucleotide, noncoding RNAs which play important roles in the regulation of gene expression at the post-transcriptional level[8]. Recently, it has been reported that specific miRNAs in blood are promising biomarkers for cancer, liver injury and heart failure [9], [10], [11]. These studies showed that the levels of specific circulating miRNAs are associated with the development of these pathological processes. It has also been reported that miRNAs are released from cells through an exosomal-mediated pathway[12], suggesting that circulating miRNAs are packaged in exosomes, which protects them from RNases.

We hypothesized that the expression levels of specific serum circulating miRNAs may be useful to monitor the pathological progression of muscular diseases, and therefore explored the possibility of these miRNAs as new biomarkers for muscular diseases. Here, we demonstrate that the serum levels of several muscle-specific miRNAs are increased in the dystrophin-deficient muscular dystrophy mouse model, *mdx*, as well as the canine X-linked muscular dystrophy in Japan dog model (CXMD_J_) [13], [14], [15]. These results suggest that serum miRNAs are useful as markers for muscular dystrophy.

## Results

**Figure 1:**
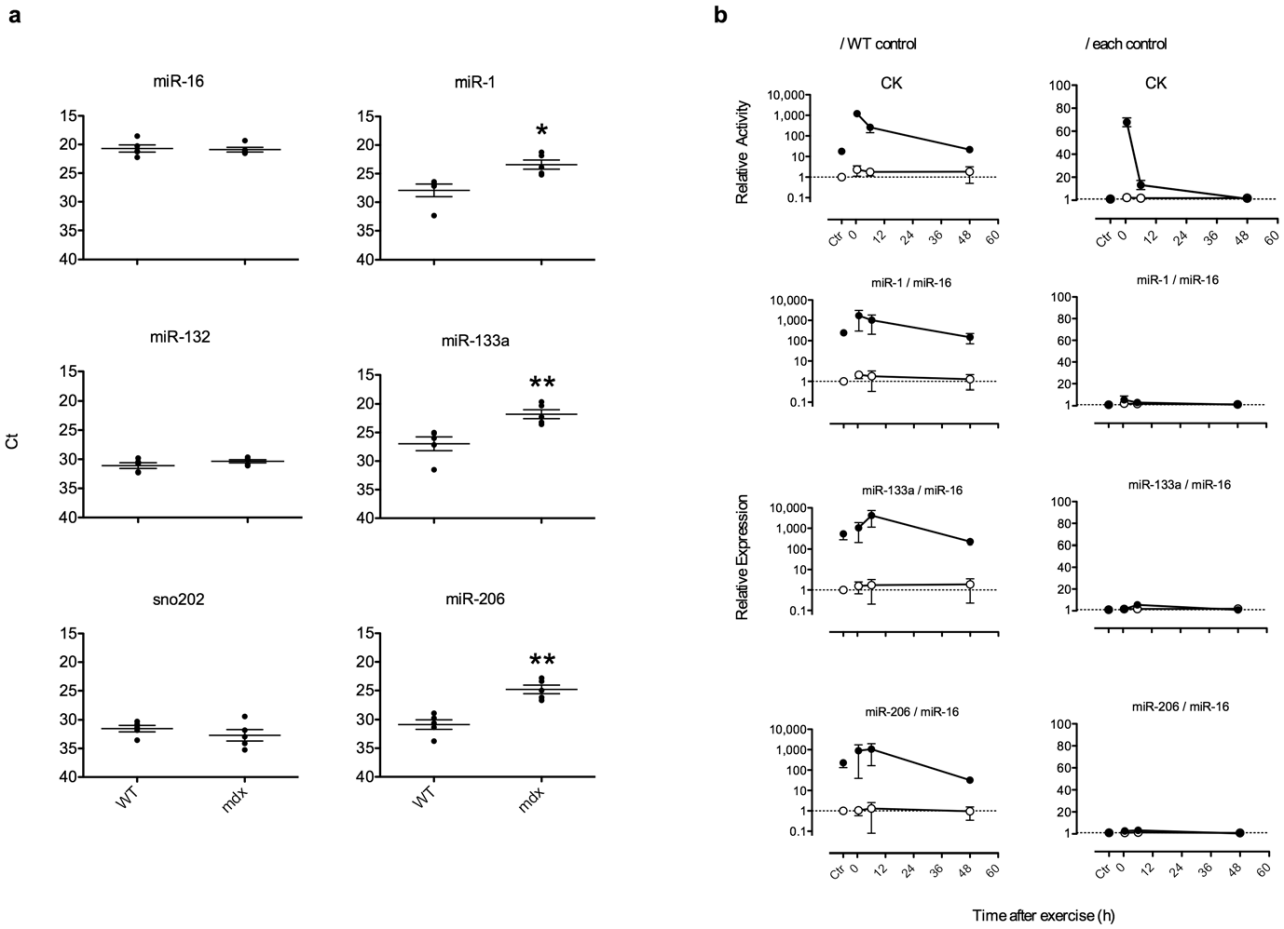
Elevation of muscle-specific miRNA levels in *mdx* mouse serum. (a) Expression levels of miRNAs in 8-week old male wild-type and *mdx* serum. Ct was determined by real-time PCR. In these graphs, the longer bars on each plot indicate the mean, and the shorter bars indicate ± SEM, n = 5. Asterisk (*) indicates a significant difference (*, P<0.05; **, P<0.01, two-tailed Student's t-test.). The actual P value for each test was P = 0.797 (miR-16), 0.222 (miR-132), 0.344 (sno202), 0.011 (miR-1), 0.007 (miR-133a) and 0.001 (miR-206). (b) CK and miRNA expression levels in wild-type and *mdx* serum after treadmill exercise. Running on the treadmill was continued for 20 min. About 100 µl of blood was collected from the tail vein at 0.5, 6 and 48 h after the exercise. Six days before the test, blood was collected as a control. Expression levels were normalized to the wild-type control (left) or each individual control (right). Data are presented as mean ± SEM, n = 3. ○: wild-type; •: *mdx*.

To explore the possibility of miRNA as a biomarker for DMD, we quantified the expression levels of several miRNAs in the serum of *mdx* by real-time PCR. The expression levels of miRNAs are indicated as either cycle threshold (Ct) (**Figure 1a**) or fold expression compared to wild-type (**Figure S1**). The Ct values of the ubiquitously expressed miR-16, brain-rich miR-132 [16] and small nucleolar RNA 202 (sno202) did not show any significant differences between wild-type and *mdx* serum (**Figure 1a**). In contrast, muscle-specific miR-1, -133a and -206 [17], [18], [19] were significantly increased in *mdx* (**Figure 1a**). The expression levels of these miRNAs in *mdx* were 10- to 100-fold higher than in wild-type controls (**Figure S1**). In Figure 1, the data are shown without normalization by an internal control RNA. Although small nuclear RNA U6, sno202 and ubiquitously expressed miRNA, such as miR-16, are often used as an internal control for miRNA analysis, there is currently no consensus for a serum internal control miRNA for real-time PCR analysis. Indeed, we examined the expression of U6 but found it was undetectable in serum (data not shown), and sno202 and miR-16 revealed no significant difference between wild-type and *mdx* (**Figure 1a** and **Figure S1**). In addition, miR-16 was more abundant than sno202 in serum (**Figure 1a**). Therefore, we employed miR-16 as the internal control for normalization of muscle-specific miRNAs in serum in the subsequent studies.

We also confirmed the accuracy of miR-16 as an internal control by using exogeneous miRNA (spiked-in miRNA). *C. elegans* miRNA-39 (cel-miR-39) was used as a spiked-in miRNA because of the lack of sequence homology to mouse miRNAs. Synthetic cel-miR-39 was spiked into serum after the addition of denaturing solution including RNase inhibitors. Then, miRNAs were isolated and the levels of cel-miR-39, miR-16, -1, -133a and -206 were determined by real-time PCR (**Figure S2**). In three-repeated experiments, the quantities of cel-miR-39 and miR-16 showed similar levels each time (**Figure S2a**). Furthermore, the expression levels of miR-1, -133a and -206 were calculated by normalization with cel-miR-39 or miR-16, individually (**Figure S2b**). The expression levels of miR-1, -133a and -206 were highly elevated in *mdx*, and the results were consistent between normalization with cel-miR-39 and miR-16.

It is conceivable that leakage or secretion from skeletal muscle fibers is the major cause of the increase in muscle-specific miRNAs in serum, but there remains the possibility that these miRNAs are excessively expressed in dystrophic skeletal muscle, which then influences serum expression levels. To investigate this possibility, we examined the expression level of these miRNAs in the skeletal muscle (soleus: Sol, tibialis anterior: TA and diaphragm: DIA) of *mdx* (**Figure S3**). Levels of ubiquitously expressed miR-16 were not different among the muscles examined, but miR-1 and miR-133a were significantly decreased in Sol and TA of *mdx*, although the differences are less than 2-fold. On the other hand, miR-206 was significantly increased in *mdx* TA and DIA, but not in Sol, and this increase of miR-206 in some *mdx* muscles could be related to a previously reported role for miR-206 in muscle regeneration[20]. Since miR-1 and -133a levels were highly elevated in *mdx* serum, although they were not increased in *mdx* skeletal muscle, suggests that the increase of muscle-specific miRNAs in *mdx* serum is caused by an increase in leakage or secretion of miRNAs from muscle.

Since it is very important to investigate whether muscle-specific miRNA levels are affected by exercise like as CK, we compared CK and miRNA levels in mice serum after exercise using a treadmill. Both CK and miRNA were increased after the treadmill exercise (**Figure 1b**, left, normalized to wild-type control), however miRNAs appeared to be less affected. When the increase in miRNAs were corrected by the data before exercise in each group (**Figure 1b**, right, normalized by each control), CK showed almost a 60-fold increase after exercise, whereas the change of muscle-specific miRNA levels was less than 10-fold.

**Figure 2:**
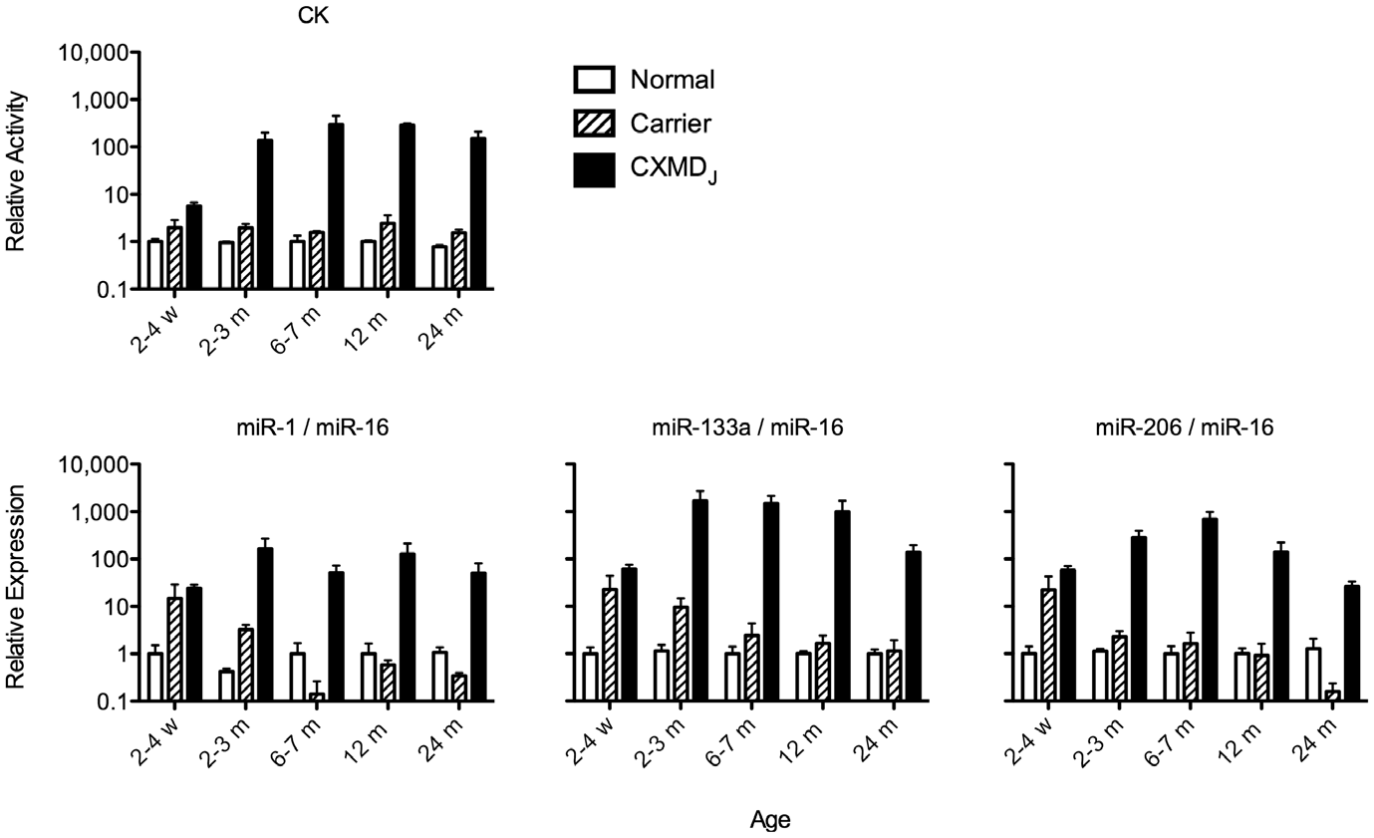
Elevation of muscle-specific miRNAs in CXMD_J_ dog serum. CK activity and miRNA expression in the serum of normal, carrier and dystrophy dogs (CXMD_J_) at the indicated ages were determined. Expression levels of miR-1, miR-133a, miR-206 and miR-16 were determined by real-time PCR, and levels of each muscle-specific miRNA (miR-1, miR-133a and miR-206) was corrected by miR-16 levels. Results are indicated as relative expression to normal dogs at each age, and are presented as mean ± SEM, n = 3. w: weeks; m: months.

CXMD_J_ is a well characterized dog model of DMD, which shows severe and progressive symptoms[13], [14], [15]. We therefore analyzed the expression levels of miRNAs in normal, carrier (females possessing a mutant *dystrophin* gene on one of two X-chromosomes) and CXMD_J_ dog serum at various ages. The Ct value of these miRNAs in CXMD_J_ was significantly smaller than age-matched controls (**Figure S4**). Relative expression levels corrected by miR-16 are shown in **Figure 2**. These miRNAs are apparently able to distinguish CXMD_J_ from age-matched normal dogs. Shimatsu *et al.* previously reported that the CK concentration of CXMD_J_ dogs do not increase with age [21]. Thus, our results of these miRNA levels are consistent with the CK levels in this model.

Our data indicate that the levels of miR-1, -133a and -206 relative to miR-16 are increased in the serum of two animal models of muscular dystrophy, *mdx* and CXMD_J_. It is very intriguing that serum miRNA were less affected by stress, such as exercise, compared with CK. In conclusion, muscle-specific miRNAs in serum may be useful biological markers for muscular dystrophy which are more reliable than CK, and further investigations are required to clarify the molecular mechanisms by which miRNAs are released from the inside of cells into serum.

## Discussion

Recently, several studies have reported that miRNAs in serum are promising biomarkers for diseases, such as cancers, liver injury or heart failure [9], [10], [11]. CK is commonly used as a biomarker of muscular diseases to evaluate the level of muscle damage and necrosis, and the efficacy of potential therapies, but it is not always reliable since it is easily affected by stress to the body, such as exercise [5], [6], [7]. Therefore, more reliable biomarkers of muscular dystrophy have long been desired. We hence investgigated whether serum miRNAs are useful for monitoring the pathological condition of muscular diseases. In this report, we demonstrate that the serum levels of several muscle-specific miRNAs are increased in two dystrophin-deficient muscular dystrophy animal models. Importantly, we show that the levels of these miRNAs are much less affected by stress to the body compared with CK levels.

To investigate the mechanism of the increase of miRNA expression, we also examined the expression level of miR-1, miR-133a and miR-206 in the skeletal muscle of *mdx* (**Figure S3**). miR-1 and -133a were significantly decreased in Sol and TA of *mdx*. On the other hand, miR-206 was significantly increased in TA and DIA of *mdx*. Our results suggest that the increase of muscle-specific miRNAs in the serum of these DMD models is caused by an increase in leakage or secretion of miRNAs from muscle, and not by the change of expression in skeletal muscle. However, it is not yet clear whether the increase of these miRNAs is caused by leakage or secretion from muscle. It is conceivable that leakage from skeletal muscle fibers is the major cause of the increase in muscle-specific miRNAs in mdx serum, but it is hard to explain why these miRNAs were not degraded by RNase. Mitchell *et al.* [10] showed that synthetic miRNAs are immediately degraded in serum even though endogenous circulating miRNAs are stably expressed in serum. To explain these results, they suggested that miRNAs are released from cells through an exosomal-mediated pathway. If circulating miRNAs are secreted by an exosomal-mediated pathway, it is possible that dystrophin is involved in the regulation of exosome secretion and a lack of dystrophin results in increased miRNA release. However, further investigation is required to clarify the contribution of dystrophin in exosome secretion.

The change of miRNA expression levels in skeletal muscle of *mdx* in this report is consistent with previous reports [20], [22]. It is intriguing that TA muscle of denervated mice also showed an increase in miR-206 and a decrease in miR-1 and -133a [23]. Yuasa *et al.* [20] also showed that miR-206 expression was increased after cardiotoxin-induced muscle regeneration and that miR-206 contributes to muscle regeneration. Interestingly, it has been showed that the expression levels of miR-206 in DMD patients are not increased [24] or that the increase is not as large as in *mdx* [22]. Although *mdx* mice are deficient in dystrophin, they do not show lethality unlike in humans. Increased miR-206 expression levels in *mdx* therefore contribute to the different phenotype between humans and mice. In addition, Williams *et al.* [23] showed that expression of miR-206 delayes disease progression and promotes regeneration of neuromuscular synapses in amyotrophic lateral sclerosis (ALS) model mice. Taken together, these results indicate that gene therapy using miR-206 may be a useful treatment for muscular diseases.

In this report, we focused on muscle-specific miRNAs and found that they are significantly increased in serum of DMD models. To investigate whether such an increase can be observed in some myopathy models which do not have any effective diagnosis markers, we also measured these muscle-specific miRNAs in serum of steroid treated dogs. We found that serum level of miR-1, -133a and -206 were not increased in steroid treated dog did not show increase compared with non-treatment controls (data not shown). Intriguingly, *Lodes et al.* [25] performed microarray analysis with circulating miRNAs and found an increase in specific miRNAs in serum of cancer patients. Furthermore the miRNA expression patterns were able to discriminate between healthy controls and cancer patients. Such a microarray analysis may be useful for identifying diagnosis markers for muscular diseases for which effective diagnosis markers currently do not exist.

## Materials and Methods

### Ethics Statement

The dog study was approved by the Ethics Committee for the Treatment of Middle-sized Laboratory Animals of the National Institute of Neuroscience, National Center of Neurology and Psychiatry, approval ID: 21-02 and 22-02. The mice study was approved by the Ethics Committee for the Treatment of Laboratory Animals of the National Institute of Neuroscience, National Center of Neurology and Psychiatry, approval ID: 2008011.

### Animals and serum samples

All animals in this study were cared for and treated in accordance with the guidelines provided by the Ethics Committee for the Treatment Laboratory Animals of National Institute of Neuroscience, or the Ethics Committee for the Treatment Laboratory Middle-sized Animals of National Institute of Neuroscience. Skilled experimental animal technicians, who have special knowledge of methods to prevent unnecessary excessive pain, handled the animals and assisted in the experiments.

As Duchenne muscular dystrophy (DMD) models, the X-chromosome-linked muscular dystrophy (*mdx*) mouse and canine X-linked muscular dystrophy in Japan (CXMD_J_), Beagle-based medium-sized dystrophic dogs, were used in this study. In **Figure 1a**, whole body blood of male *mdx* mice (n = 5) or age-matched controls (strain C57BL/10; B10) (n = 5) at 8 weeks were collected from the abdominal vena cava under anesthesia. Blood collection after the treadmill test (**Figure 1b**), was performed from the tail vein of *mdx* (n = 3) or age-matched control (n = 3) under anesthesia. The phenotype of CXMD_J_ has been reported previously[13], [14], [15]. For analysis of the serum of CXMD_J_ dogs, mutation carrier female dogs and wild-type control dogs (n = 3, at each age indicated in Figure 2), blood was collected from the subcutaneous vein of the hindlimb, and whole blood was allowed to stand for about 1 h at room temperature before centrifugation at 1,800 g for 10 min at room temperature. The resultant serum was dispensed into a 1.5 ml cryotube and stored at −80°C until use.

### RNA isolation and quantification of miRNA

Total RNA, including miRNA, was extracted from 50 µl of serum using the mirVana miRNA isolation kit (Ambion, Austin, TX, USA) according to the manufacturer's instructions, and finally eluted with 50 µl of elution buffer provided by the manufacturer. Five µl of total RNA was reverse transcribed using the TaqMan miRNA Reverse Transcription kit (Applied Biosystems, Foster City, CA, USA) and miRNA-specific stem-loop primers (part of TaqMan miRNA assay kit; Applied Biosystems). The expression levels of miRNA were quantified by real-time PCR using individual miRNA-specific primers (part of TaqMan miRNA assay kit; Applied Biosystems) with 7900HT Fast Real-Time PCR System (Applied Biosystems) according to the manufacturer's instructions. There is no current consensus on the use of an internal control for real-time PCR analysis of serum miRNA. Therefore, we used fixed volumes of starting serum (50 µl), buffer for the elution of RNA (50 µl) from starting serum, and input into the RT reaction (5 µl) in each assay for technical consistency. Data analysis was performed by SDS 2.1 real-time PCR data analysis software (Applied Biosystems). Threshold was fixed at 0.2 in each analysis for data consistency. The similarities of linearity of primers for each target miRNA were confirmed by using a dilution series of synthetic miRNAs.

### Spiked-in miRNA experiment

We followed the protocol previously reported by Mitchell *et al*. [10] to determine endogenous miRNA levels with spiked-in miRNA. Spiked-in miRNA was designed against *C. elegans* microRNA-39 (cel-miR-39)(5′-UCACCGGGUGUAAAUCAGCUU-3′), and was synthesized by Sigma Aldrich Japan. Synthetic cel-miR-39 was spiked into serum after the addition of denaturing solution including RNase inhibitors. Isolation of total RNA, including miRNA, and quantification of the expression levels of miRNAs by real-time PCR were performed as described above.

### Creatine kinase determination

Serum creatine kinase (CK) levels were assayed with the Fuji Drychem system (Fuji Film Medical Co. Ltd, Tokyo, Japan) according to the manufacturer's instructions. Data was expressed as units per liter (U/l).

### Treadmill test

Mice were forced to run on a treadmill (MK-680S treadmill: Muromachi Kikai, Tokyo, Japan) with an inclination of 0° at 5 m/min for 5 min. Then, the speed was increased by 1 m/min every minute for a further 15 min. After the running, blood was immediately collected from the tail vein, as well as subsequently collected at the indicated times.

### Statistics

Statistical significances between groups were determined by the two-tailed t-test, or one-way ANOVA with Bonferroni post hoc test. Each analysis was performed by Prism 5 (Graphpad Software Inc., San Diego, CA, USA).

## Supporting Information

Figure S1 **miRNA expression in 8-week old male wild-type control and** ***mdx*** **serum.** Expression levels of miRNAs were determined by real-time PCR. Results are shown as relative expression, and data are presented as mean ± SEM, n = 5.
(TIFF)

Figure S2 **(a) Confirmation of the consistency of miRNA isolation from serum.** *C. elegans* miR-39 (cel-miR-39) was chemically synthesized and added to the denatured mouse serum samples. Total RNA was isolated from the mouse serum samples, and the quantity of exogenous cel-miR-39 and endogenous miR-16 were determined by real-time PCR. (b) Expression levels of miR-1, -133a and -206 in wild-type control and *mdx* serum, which were individually normalized by the cel-miR-39 spiked-in control or the endogenous control, miR-16. Results are shown as relative expression. The longer bars on each plot indicate the mean, and the shorter bars indicate ± SEM, n = 3.
(TIFF)

Figure S3 **miRNA expression in wild-type control and** ***mdx*** **muscles.** Expression levels of miR-1, -16, -133a and -206 in Soleus (Sol), tibialis anterior (TA) and diaphragm (DIA) were determined by real-time PCR. Results are shown as relative expression. sno202 was used as an internal control. Data are presented as mean ± SEM, n = 4. Asterisk (*) indicates a significant difference (*, P<0.05; P<0.01, two-tailed Student's t-test.). The actual P value for each test was P = 0.024 (miR-1) and 0.010 (miR-206) in Sol; P = 0.002 (miR-1), 0.008 (miR-133a) and <0.001 (miR-206) in TA; P = 0.006 (miR-206) in DIA.
(TIFF)

Figure S4 **Expression levels of muscle-specific miRNAs in the serum of normal, carrier and dystrophy dogs (CXMD_J_) at the indicated ages.** Each Ct was determined by real-time PCR. In these graphs, the longer bars on each plot indicate the mean, and the shorter bars indicate ± SEM, n = 3. Asterisk (*) and pound (#) indicate a significant difference (*, P<0.05; **, P<0.01; ***, P<0.001 from normal: #, P<0.05; ##, P<0.01; ###, P<0.001 from carrier, one-way ANOVA with Bonferroni post hoc test). w: weeks; m: months.
(TIFF)
