# Supplementary figures and images for: Identification of Muscle-Specific MicroRNAs in Serum of Muscular Dystrophy Animal Models: Promising Novel Blood-Based Markers for Muscular Dystrophy
Source: PLoS One. 2011 Mar 30;6(3):e18388. doi: 10.1371/journal.pone.0018388 (PMC3068182; doi:10.1371/journal.pone.0018388)

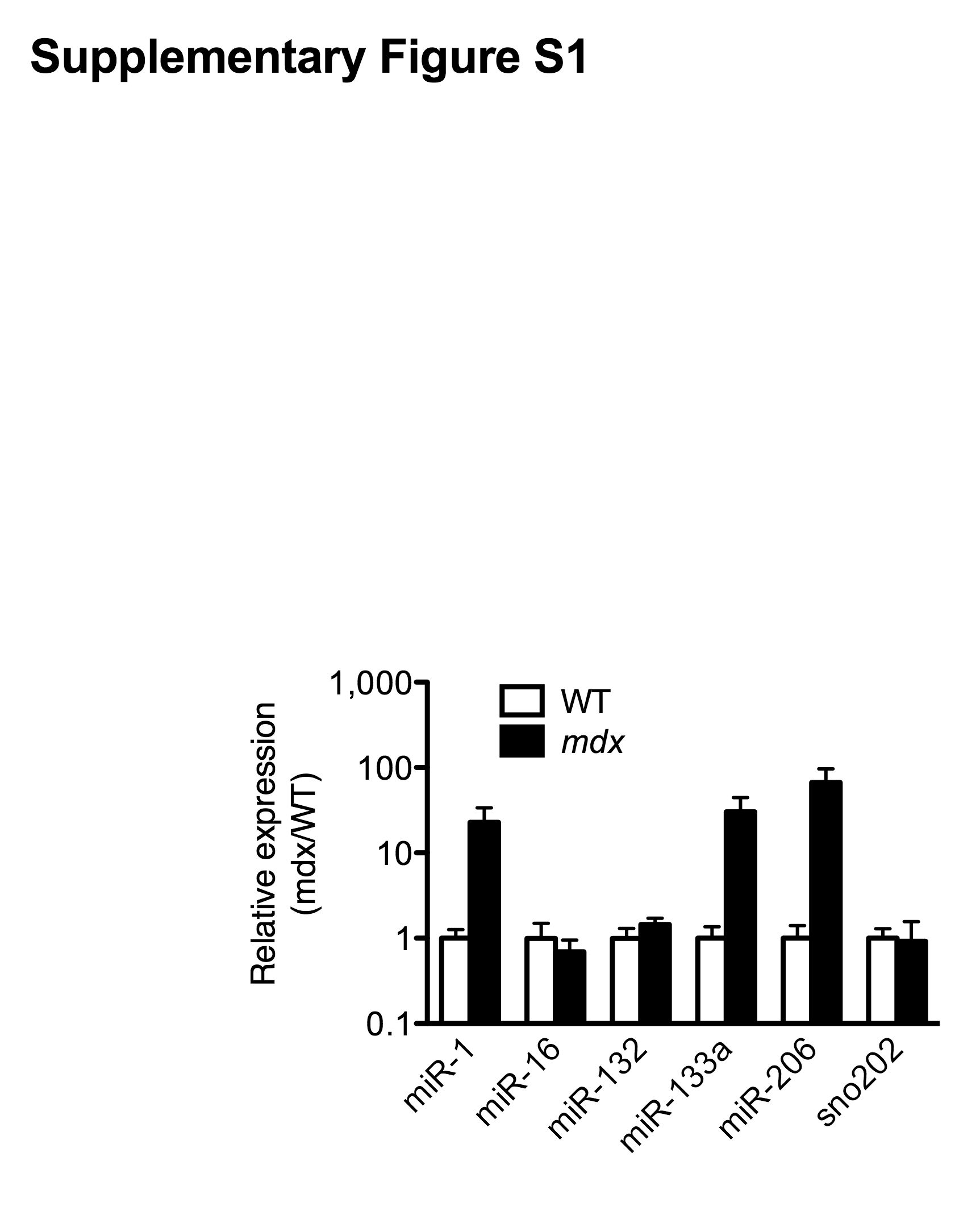

Supplement: Figure S1 — miRNA expression in 8-week old male wild-type control and mdx serum. Expression levels of miRNAs were determined by real-time PCR. Results are shown as relative expression, and data are presented as mean ± SEM, n = 5. (TIFF) [file pone.0018388.s001.tiff]

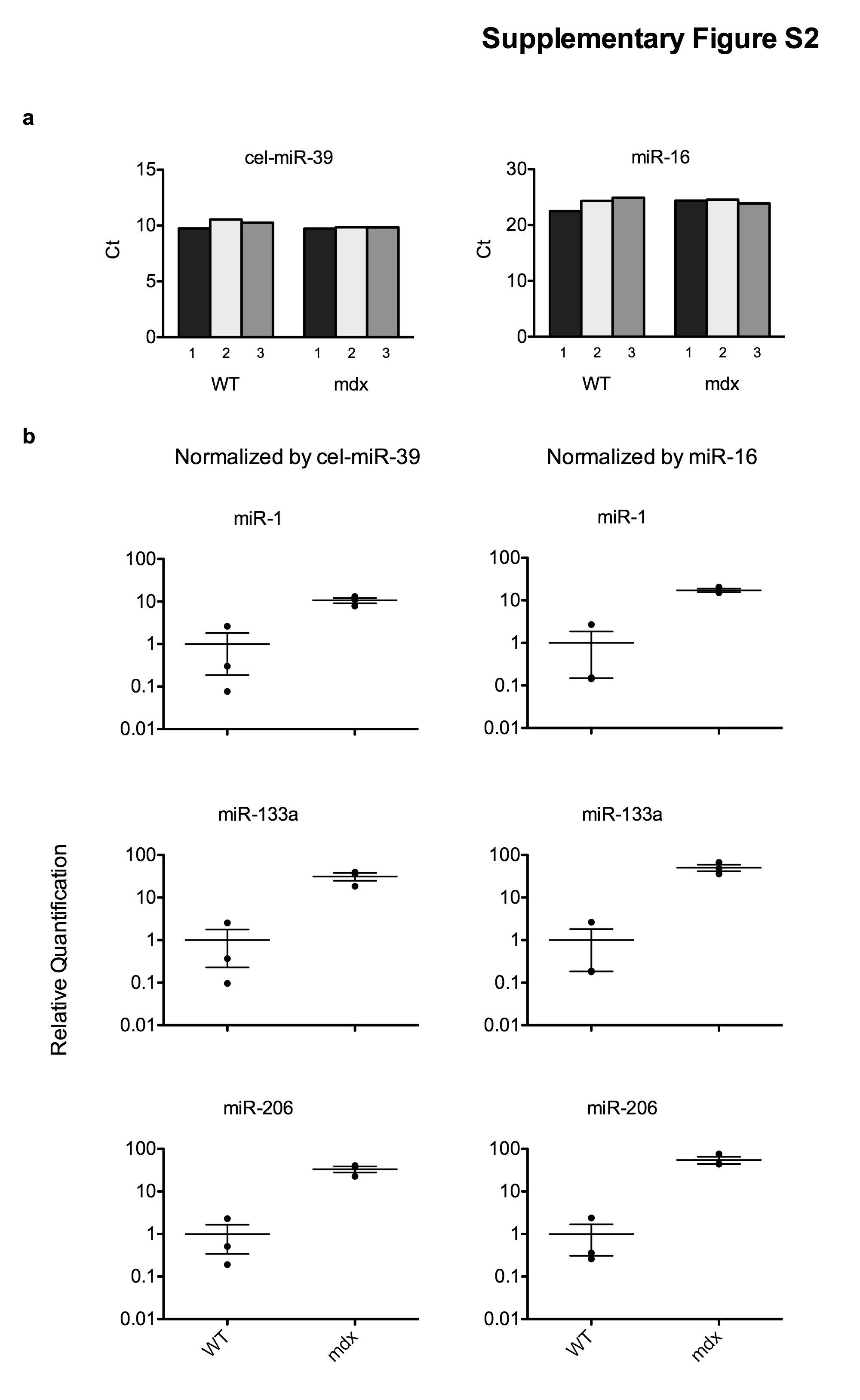

Supplement: Figure S2 — (a) Confirmation of the consistency of miRNA isolation from serum. C. elegans miR-39 (cel-miR-39) was chemically synthesized and added to the denatured mouse serum samples. Total RNA was isolated from the mouse serum samples, and the quantity of exogenous cel-miR-39 and endogenous miR-16 were determined by real-time PCR. (b) Expression levels of miR-1, -133a and -206 in wild-type control and mdx serum, which were individually normalized by the cel-miR-39 spiked-in control or the endogenous control, miR-16. Results are shown as relative expression. The longer bars on each plot indicate the mean, and the shorter bars indicate ± SEM, n = 3. (TIFF) [file pone.0018388.s002.tif]

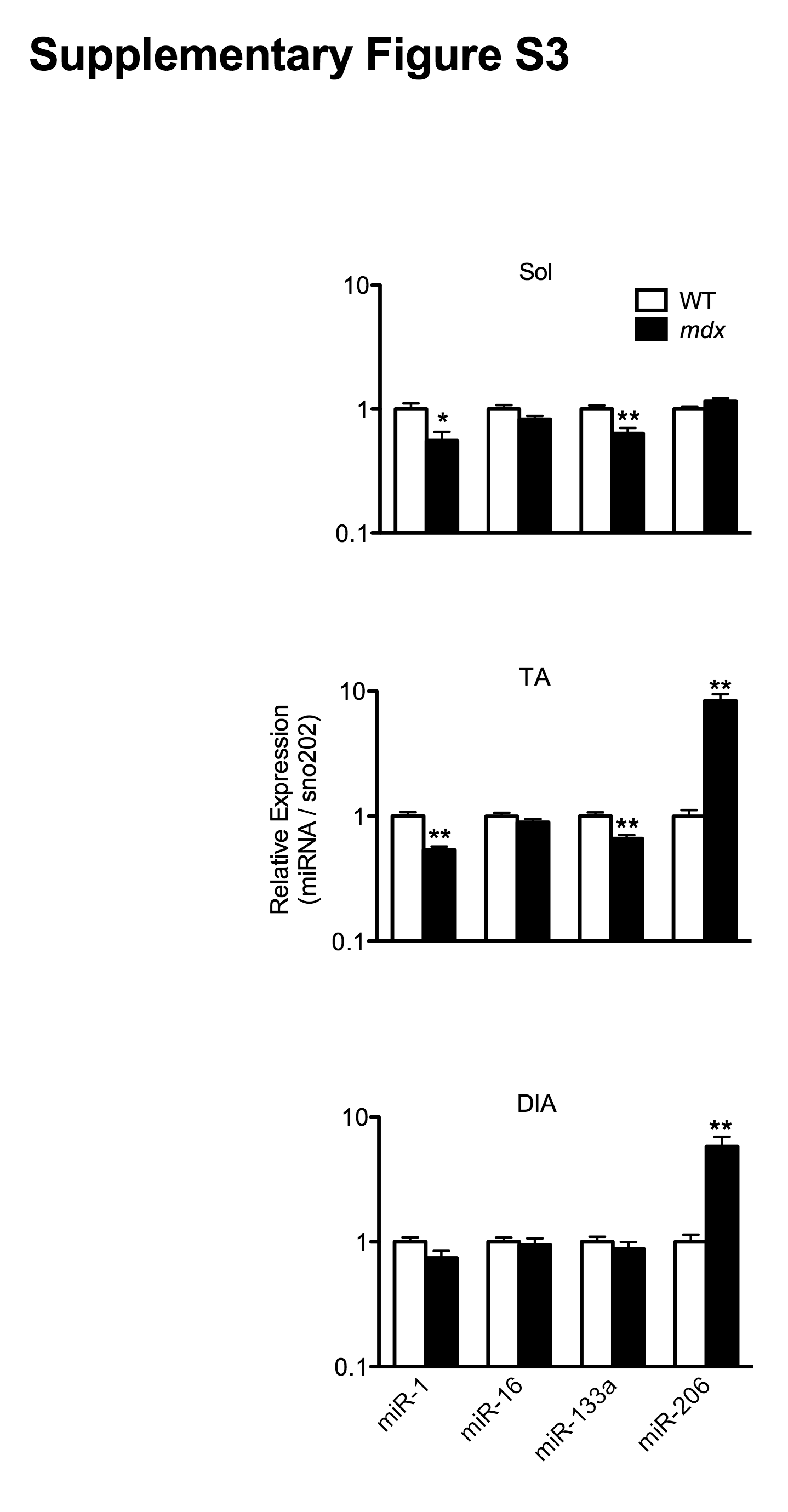

Supplement: Figure S3 — miRNA expression in wild-type control and mdx muscles. Expression levels of miR-1, -16, -133a and -206 in Soleus (Sol), tibialis anterior (TA) and diaphragm (DIA) were determined by real-time PCR. Results are shown as relative expression. sno202 was used as an internal control. Data are presented as mean ± SEM, n = 4. Asterisk (*) indicates a significant difference (*, P<0.05; P<0.01, two-tailed Student's t-test.). The actual P value for each test was P = 0.024 (miR-1) and 0.010 (miR-206) in Sol; P = 0.002 (miR-1), 0.008 (miR-133a) and <0.001 (miR-206) in TA; P = 0.006 (miR-206) in DIA. (TIFF) [file pone.0018388.s003.tiff]

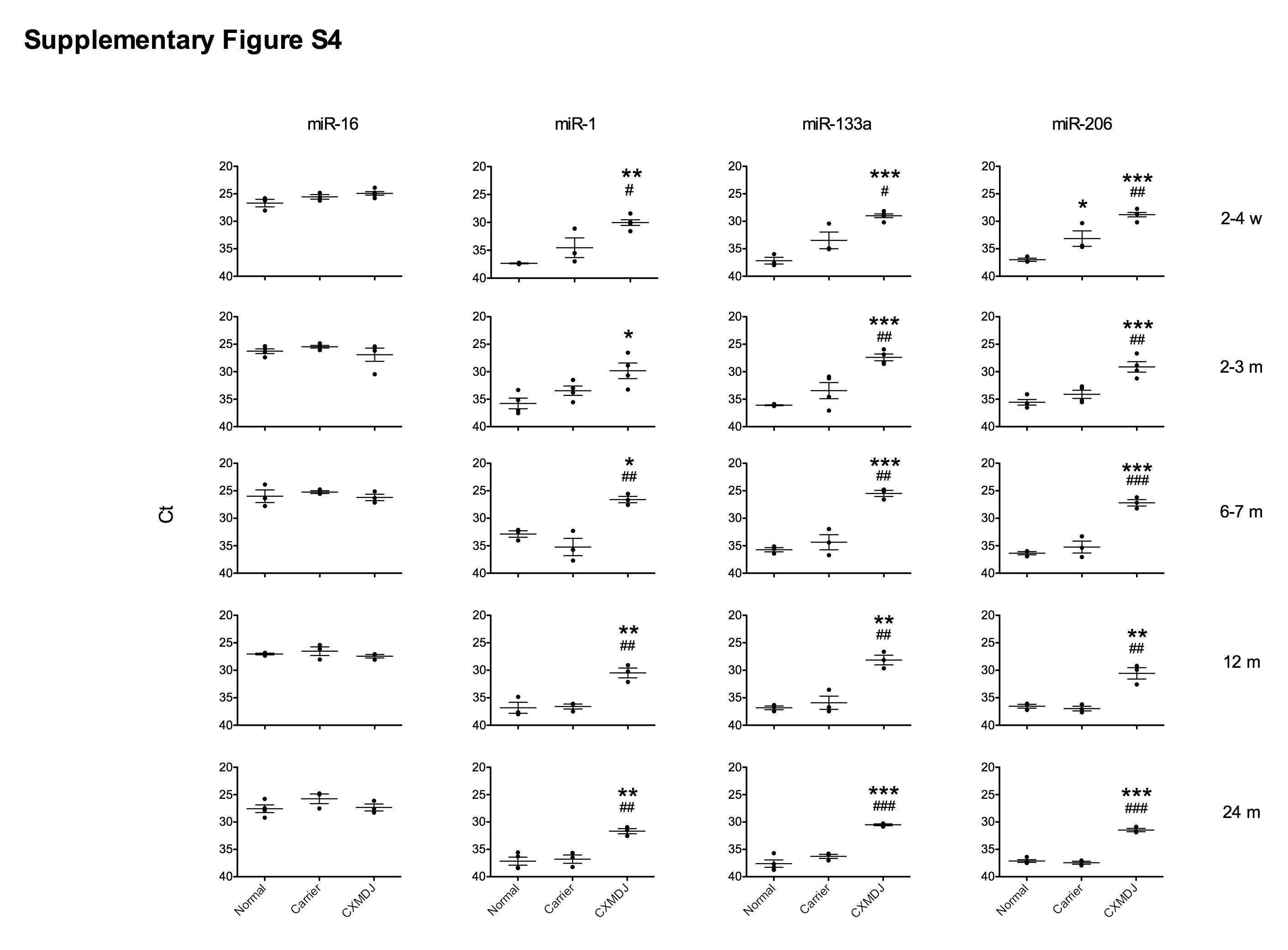

Supplement: Figure S4 — Expression levels of muscle-specific miRNAs in the serum of normal, carrier and dystrophy dogs (CXMDJ) at the indicated ages. Each Ct was determined by real-time PCR. In these graphs, the longer bars on each plot indicate the mean, and the shorter bars indicate ± SEM, n = 3. Asterisk (*) and pound (#) indicate a significant difference (*, P<0.05; **, P<0.01; ***, P<0.001 from normal: #, P<0.05; ##, P<0.01; ###, P<0.001 from carrier, one-way ANOVA with Bonferroni post hoc test). w: weeks; m: months. (TIFF) [file pone.0018388.s004.tiff]
